# Pharmacogenomics in the era of personalised medicine

**DOI:** 10.5694/mja2.51759

**Published:** 2022-10-18

**Authors:** Cassandra White, Rodney Scott, Christine L Paul, Stephen P Ackland

**Affiliations:** ^1^ Maitland Hospital Maitland NSW; ^2^ University of Newcastle Newcastle NSW; ^3^ Pathology North Newcastle NSW; ^4^ Priority Research Centre for Health Behaviour University of Newcastle Newcastle NSW; ^5^ Lake Macquarie Private Hospital Gateshead NSW; ^6^ Hunter Cancer Research Alliance Newcastle NSW

**Keywords:** Pharmacogenomics, Cancer, Genetic testing, Prescribing

Australia should develop a sustainable evidence‐based pharmacogenomic screening program, with *DPYD* genotyping at the forefront

Pharmacogenomics is the genomic profiling of patients for genetic variants that clinically modify the tolerability and desired effect of specific medications. Patients who carry functional gene variants may have increased or decreased capacity to metabolise medications upon exposure. Some genetic variants, such as those occurring in the cytochrome P450 (*CYP)* genes, have an impact on multiple medications across different drug classes. Other gene–drug pairs are more specific, such as uridine diphosphate glucuronosyltransferase 1A1 (*UGT1A1*)*–*irinotecan or dihydropyrimidine dehydrogenase (*DPYD*)–fluoropyrimidine chemotherapy.^1^ Pharmacogenomics represents a multidisciplinary collaborative endeavour, including both clinical pharmacology and clinical pathology disciplines as well as various clinicians, with the shared goal of improving health care delivery for patients through individualisation of prescribing and patient care.^2^, ^3^, ^4^

There are many known gene–drug pairs across various drug classes, including immunosuppressants, psychotropics, antimicrobials, antidepressants, selective oestrogen receptor modulators, beta blockers, statins, proton pump inhibitors, anticoagulants, and antiplatelet agents.^5^ Gene variant frequencies differ substantially between genes (4–74%),^6^ as does the severity of drug toxicity.^6^ Patients who poorly metabolise medications face toxicities ranging from moderate to severe, requiring hospitalisation or intensive care unit admission and even resulting in death. Testing for genomic variants implicated in the tolerability of select medications can help us to predict a patient's metabolic response and adjust medications accordingly, allowing for individualised prescribing.^3^, ^7^

Not all gene–drug pairs carry sufficient evidence to warrant adjustments in clinical prescribing. Additionally, not all medication toxicity is explainable through the identification of gene variants; there are several up‐ and downstream regulators that effect the pharmacokinetic and pharmacodynamic impacts of therapeutic agents. The clinical introduction of a pharmacogenomics strategy to personalise drug selection and/or dosing requires an assessment of the cost‐utility and cost‐effectiveness of implementation in the Australian health care system in each case.^3^ Gene–drug pairs where the cost‐effectiveness is marginal or negative may not be worth implementing, such as when the genotyping is too expensive or time‐consuming, variant frequency is low, the clinical implications of inadvertent toxicity are not significant, or there are simpler ways to avoid serious toxicity. Rigorous evaluation of pharmacogenomic evidence has informed pharmacogenomics‐guided prescribing guidelines developed by collaborative groups, including the Clinical Pharmacogenetics Implementation Consortium and the Dutch Pharmacogenetics Working Group (Box 1).^1^, ^8^

Box 1 Gene–drug pairs, in accordance with evidence‐based dose adjustment guidelines*

**Table:** 

Drug class	Drug	Gene
Anticancer agents and immunosuppressants	Azathioprine	*TPMT*
	Mercaptopurine	*TPMT*
	5‐Fluorouracil	*DPYD*
	Capecitabine	*DPYD*
	Irinotecan^†^	*UGT1A1*
	Tacrolimus	*CYP3A5*
	Tamoxifen	*CYP2D6*
Anticoagulants and antiplatelet agents	Warfarin	*CYP2C9, VKORC1, CYP4F2*
	Clopidogrel	*CYP2C19*
Antimicrobials	Abacavir	*HLA‐B*
	Voriconazole	*CYP2C19*
	Aminoglycosides^‡^	*MT‐RNR1*
Antidepressants	Fluvoxamine^‡^	*CYP2D6, CYP2C19*
	Citalopram	*CYP2D6, CYP2C19*
	Nortriptyline	*CYP2D6, CYP2C19*
	Amitriptyline	*CYP2D6, CYP2C19*
	Sertraline	*CYP2D6, CYP2C19*
Anticonvulsants	Carbamazepine^‡^	*HLA‐A, HLA‐B*
	Phenytoin	*HLA‐B, CYP2C9*
Statins	Simvastatin^‡^	*SLCO1B1, ABCG2, CYP2C9*
	Atorvastatin^‡^	*SLCO1B1, ABCG2, CYP2C9*
	Rosuvastatin^‡^	*SLCO1B1, ABCG2, CYP2C9*
Proton pump inhibitors	Omeprazole	*CYP2C19*
	Pantoprazole	*CYP2C19*
	Lansoprazole	*CYP2C19*
Anti‐gout agents	Allopurinol^‡^	*HLA‐B*
	Rasburicase^‡^	*G6PD*
Analgesics	Opioids^‡^	*COMT, CYP2D6, OPRM1*
	NSAIDs	*CYP2C9*

NSAIDs = non‐steroidal anti‐inflammatory drugs.

* Adapted from Clinical Pharmacogenetics Implementation Consortium^1^ and Dutch Pharmacogenetics Working Group^8^ guidelines. The table includes genes implicated in both drug metabolism and hypersensitivity reactions where known gene–drug pairs are identified and dose adjustment guidelines are available. Full individual gene names are listed within the relevant dose adjustment guidelines. Please note this list not exhaustive.

† Dutch Pharmacogenetic Working Group guidelines^8^ only.

‡ Clinical Pharmacogenetics Implementation Consortium guidelines^1^ only.

An example of successful international implementation is the introduction of upfront *DPYD* genotyping in patients intended to receive fluoropyrimidine chemotherapy (5‐fluorouracil and capecitabine).^9^ Globally, more than 2 million cancer patients receive fluoropyrimidine per annum, including approximately 10 000 patients in Australia.^10^ Serious grade 3 and 4 toxicities^11^ resulting in hospitalisation and occasional intensive care admission occur in approximately 30% of treated cases.^7^, ^12^ Up to 1% of patients die from fluoropyrimidine chemotherapy‐related toxicity.^7^ Four *DPYD* variants are found in approximately 3–8% of the European population and lead to a significant increase in the relative risk of developing ≥ grade 3 adverse events including diarrhoea, mucositis, myelosuppression, and palmar‐plantar erythrodysaesthesia (Box 2).^13^ There is evidence to support reducing fluoropyrimidine dosing in patients carrying significant *DPYD* gene variants, leading to decreased toxicity without compromising treatment response.^14^, ^15^, ^16^, ^17^ Clinical Pharmacogenetics Implementation Consortium and Dutch Pharmacogenetics Working Group guidelines for dose modification provide tailored instructions for each significant *DPYD* variant based on the degree of expected metabolic impairment.^7^, ^12^ The clinical implications for *DPYD* variant carriers are significant and the cost burden related to management is high, despite the low frequency of *DPYD* variants compared with some other gene–drug pairs.^6^ Early prospective data and health economic modelling illustrate the cost‐effectiveness of upfront *DPYD* genotyping across various health systems internationally, and upfront *DPYD* genotyping has become part of the standard of care in the Netherlands, France, Switzerland, Germany, and the United Kingdom.^18^, ^19^, ^20^, ^21^, ^22^, ^23^, ^24^, ^25^, ^26^ Although there is limited information on the international uptake of testing, for pharmacogenomics generally and *DPYD* specifically, increases in uptake of *DPYD* genotyping rose by 14% following endorsement of pre‐emptive screening by the European Medicines Agency in 2020, showing the impact of both clinical champions and early adopters on the implementation of health practices.^26^, ^27^ To date, pre‐emptive *DPYD* screening has not been adopted systematically in Australia.

Box 2 ***DPYD* variant specific pharmacogenomic information and fluoropyrimidine (FP) dose adjustment recommendations in single heterozygote carriers**

**Table:** 

*DPYD* variant	Allele frequency	Toxicity: standard FP dose, RR (95% CI)^16^, ^17^	Metaboliser status^12^ ^,^ †	Dose instructions^12,^ ‡	Toxicity: reduced FP dose, RR (95% CI)^17^
c.1905 + 1G > A	0.8%	2.85 (1.75–4.62)	Poor	Reduce FP by 50%, monitor for clinical tolerance	1.31 (0.63–2.73)
c.1679T > G	0.06%	4.40 (2.08–9.30)	Poor	Reduce FP by 50%, monitor for clinical tolerance	No toxicity
c.2846A > T	0.4%	3.02 (2.22–4.10)	Intermediate	Reduce FP by 25–50%, monitor for clinical tolerance	2.0 (1.19–3.34)^§^
c.1236G > A	2.3%	1.72 (1.22–2.42)	Intermediate	Reduce FP by 25–50%, monitor for clinical tolerance	1.69 (1.18–2.42)^§^

RR = relative risk.

* European population data.^12^

† Intermediate metaboliser: gene activity score (GAS), 1–1.5; poor metaboliser: GAS, 0–0.5. Henricks et al^13^ assigned metabolic activity (0 = no function, 0.5 = partial function, 1 = normal function) to specific variant alleles and the cumulative score of both alleles indicates total GAS (maximum score, 2).

‡ Dose instructions for single heterozygote carriers only. § Dose reduction recommendation was 25%;^17^ subsequently updated to 25–50% in light of limited effect on RR (https://cpicpgx.org/guidelines/guideline‐for‐fluoropyrimidines‐and‐dpyd/ [viewed July 2022]).

Why has Australia continued to accept such a guarded approach to upfront pharmacogenomic screening? A position statement released by the Royal College of Pathologists of Australasia suggests that in 2017, 1.7 million Australians were prescribed drugs with known gene–drug pairs, and up to 40% of patients carry an actionable variant.^4^ Limited pharmacogenomic testing is available currently in Australia, both as gene panel screening and targeted genotyping, predominantly through private services at present.^3^, ^28^ Medicare rebate currently only applies for thiopurine methyltransferase (*TPMT*) and human leukocyte antigen (HLA‐B*57:01) genotyping for eligible patients; all others are conducted at patient expense. Currently, patients can obtain pharmacogenomic testing services through their physician or pharmacist, or directly through direct‐to‐consumer services.^28^ As a result, it is difficult to capture the extent of uptake of pharmacogenomic testing in Australia. This raises several concerns, predominantly the lack of standardisation of testing, protection of patients’ genomic information, and the interpretation and use of genomic results outside of the health care setting.^3^, ^4^, ^28^

In 2008, the Australian Centre for Health Research predicted $2.5–6.2 billion could be saved in avoiding adverse events and pharmaceutical waste related to avoidable pharmacogenomics‐guided prescribing decisions over a 5‐year projection period, estimating a cost of $14 027 per hospital admission.^29^ Further financial benefit could be expected, considering the falling cost of genomic testing, compared with the relatively stagnant cost of inpatient hospital management.

Aside from cost‐effectiveness, additional expected benefits include improved patient tolerance of drugs and subsequent improved compliance.^23^ It can be inferred that better compliance leads to better management outcomes for patients (disease control, relapse/recurrence of disease, and even overall survival). Perceived barriers include limited education and training for physicians in ordering, interpreting and applying pharmacogenomic testing prior to treatment decisions, limited staffing resources and funding for laboratory testing, limited technological support to successfully facilitate delivery of results, and historically slow turnaround times that are inadequate to provide information before critical prescribing decisions, such as chemotherapy, antimicrobials and immunosuppression.^2^, ^6^, ^29^ Understanding these barriers and developing strategies to address these and other issues for inclusion in the infrastructure of a pharmacogenomic screening program is imperative to the success of implementation.^3^ Further, as with several gene polymorphism–drug pairs, there are *DPYD* variants that illustrate differing allelic frequencies across ethnicities.^30^ This must also be considered in the tailoring of an Australian pharmacogenomic screening program, accounting for our rich ethnic diversity, and aligning with the ethical and social expectations of managing genomic data.^3^, ^30^

Australia must expand the implementation of pharmacogenomic screening to elevate the quality of care we offer to patients, on par with or better than international standards. We propose using *DPYD* genotyping as a leading example. Despite the knowledge that a critical *DPYD* variant can lead to lethal toxicity on exposure to fluoropyrimidine chemotherapies, there is still no nationally funded or endorsed support to pre‐emptively identify these individuals. There are already robust fluoropyrimidine dose guidelines and infrastructure to genotype patients through commercial laboratories. We have feasibility data confirming a median turnaround time of 7 days, although this is yet to be evaluated on a large scale and within public hospital service laboratories.^31^ Additionally, although we have some understanding of the international economic impact of *DPYD* genotyping, an Australian‐specific cost‐effectiveness analysis must be conducted to confirm financial viability. Streamlining pharmacogenomic screening through clinical stakeholders would additionally serve to improve the appropriate dissemination and interpretation of results while protecting genomic information and ensuring that prescribing guidelines are followed safely and appropriately.^3^, ^4^, ^28^ Providing Australia‐wide equitable access to pharmacogenomic services is a major challenge, but a critical one for our nation.^3^, ^4^

*DPYD* genotyping could readily serve as a prototype for the streamlined expansion of pharmacogenomic screening for utilisation across various drug classes prescribed within various medical disciplines.

## Competing interests

No relevant disclosures.

## Provenance

Not commissioned; externally peer reviewed.

## Open access

Open access publishing facilitated by The University of Newcastle, as part of the Wiley ‐ The University of Newcastle agreement via the Council of Australian University Librarians.
